# Digital phenotype of mood disorders: A conceptual and critical review

**DOI:** 10.3389/fpsyt.2022.895860

**Published:** 2022-07-26

**Authors:** Redwan Maatoug, Antoine Oudin, Vladimir Adrien, Bertrand Saudreau, Olivier Bonnot, Bruno Millet, Florian Ferreri, Stephane Mouchabac, Alexis Bourla

**Affiliations:** ^1^Service de Psychiatrie Adulte de la Pitié-Salpêtrière, Institut du Cerveau (ICM), Sorbonne Université, Assistance Publique des Hôpitaux de Paris (AP-HP), Paris, France; ^2^iCRIN (Infrastructure for Clinical Research in Neurosciences), Paris Brain Institute (ICM), Sorbonne Université, INSERM, CNRS, Paris, France; ^3^Department of Psychiatry, Sorbonne Université, Hôpital Saint Antoine-Assistance Publique des Hôpitaux de Paris (AP-HP), Paris, France; ^4^Département de Psychiatrie de l’Enfant et de l’Adolescent, Assistance Publique des Hôpitaux de Paris (AP-HP), Sorbonne Université, Paris, France; ^5^CHU de Nantes, Department of Child and Adolescent Psychiatry, Nantes, France; ^6^Pays de la Loire Psychology Laboratory, Nantes, France; ^7^INICEA Korian, Paris, France

**Keywords:** mood disorders, digital phenotyping, machine learning, artificial intelligence, depressive disorder, bipolar disorder

## Abstract

**Background:**

Mood disorders are commonly diagnosed and staged using clinical features that rely merely on subjective data. The concept of digital phenotyping is based on the idea that collecting real-time markers of human behavior allows us to determine the digital signature of a pathology. This strategy assumes that behaviors are quantifiable from data extracted and analyzed through digital sensors, wearable devices, or smartphones. That concept could bring a shift in the diagnosis of mood disorders, introducing for the first time additional examinations on psychiatric routine care.

**Objective:**

The main objective of this review was to propose a conceptual and critical review of the literature regarding the theoretical and technical principles of the digital phenotypes applied to mood disorders.

**Methods:**

We conducted a review of the literature by updating a previous article and querying the PubMed database between February 2017 and November 2021 on titles with relevant keywords regarding digital phenotyping, mood disorders and artificial intelligence.

**Results:**

Out of 884 articles included for evaluation, 45 articles were taken into account and classified by data source (multimodal, actigraphy, ECG, smartphone use, voice analysis, or body temperature). For depressive episodes, the main finding is a decrease in terms of functional and biological parameters [decrease in activities and walking, decrease in the number of calls and SMS messages, decrease in temperature and heart rate variability (HRV)], while the manic phase produces the reverse phenomenon (increase in activities, number of calls and HRV).

**Conclusion:**

The various studies presented support the potential interest in digital phenotyping to computerize the clinical characteristics of mood disorders.

## Introduction

The diagnosis of mood disorders currently relies purely on clinical interviews based on the identification of symptoms that can be either subjective (sadness, anhedonia, exaltation, etc.), or that could potentially be objectified (attention disorders, psychomotor retardation, sleep disorders, etc.). The search for biomarkers is one of the major challenges in this field, and the concept of the “Digital Phenotype” (DP) of a pathology can be understood as a kind of digital biomarker, sustaining the extended phenotype, a concept introduced by Richard Dawkins that implies that phenotypes should not be limited just to biological processes (1).

Defined in 2015 by S. H. Jain (2) and shortly after by J. Torous (3) for psychiatry, the digital phenotype refers to the real-time capture by computerized measurement tools of certain characteristics specific to a psychiatric disorder. Some behaviors or symptoms could be quantifiable, which would bring a shift in the assessment of psychiatric semiology, offering a new branch of investigation constituted by an “e-semiology.” An accelerometer or an actigraphic device can detect changes in a motor symptomatology (e.g., acceleration during a manic episode, decreased activation during a depressive episode, or replacement of graphorrhoea by an increase in the number of SMS messages sent). Models based on these new signs are starting to emerge since the miniaturization of sensors and the ubiquitous use of smartphones allow extensive data collection to which psychiatrists did not have access before.

**FIGURE 1:**
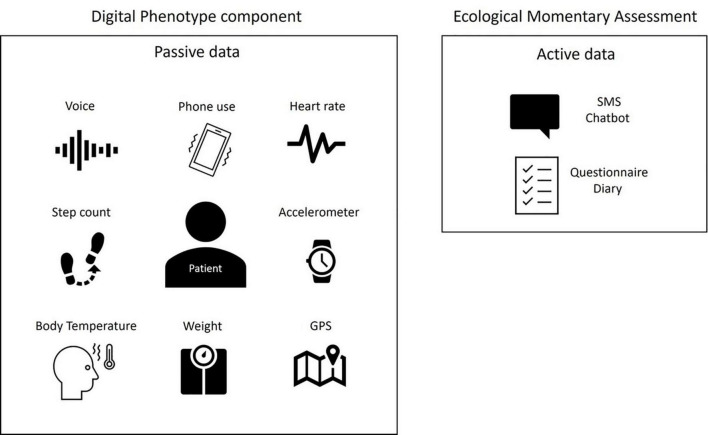
The concept of digital phenotype for mood disorders as opposed to EMA.

To highlight objective symptoms of mood disorders, the most often studied criteria are those relating to motor aspects (slowing down and restlessness), speech characteristics (speed, prosody, tenor), or sleep disorders (insomnia or hypersomnia) as well as biometric data detectable by sensors [heart rate (HR), temperature, etc.]. This collection method is called “passive data gathering”; no intervention is necessary, reducing the weight of the observers and mitigating cognitive bias of the clinicians. This term is opposed to “active data gathering,” which requires the involvement of the patient in the collection of the data (e.g., Ecological Momentary Assessment EMA) (Figure 1).

Passive data are collected automatically in real time, without requiring any input from the user and relying on tools such as the accelerometer (number of steps, motor behavior), GPS, mobile phone-based software sensing (e.g., sleep analysis), or wearables that measure HR, heart rate variability (HRV), galvanic skin conductance, temperature, blood pressure or others indicators that could be considered as potential biomarkers of certain psychiatric disorders. Smartphone use (e.g., number of SMS messages, call log, voice analysis, social media posts, internet use, online shopping, music, pictures, calendar) provides access to psychosocial functioning and to passive assessment of content.

All that information can be considered “big data” since it comes from multiple sources (e.g., multimodal passive data) and aggregates different features, and many research teams aim to determine the digital phenotypes of several mood disorders using machine learning, regression analysis or natural language processing approaches.

In this review we propose to investigate the digital phenotype of mood disorders (depressive disorder and bipolar disorder).

## Methods

**FIGURE 2:**
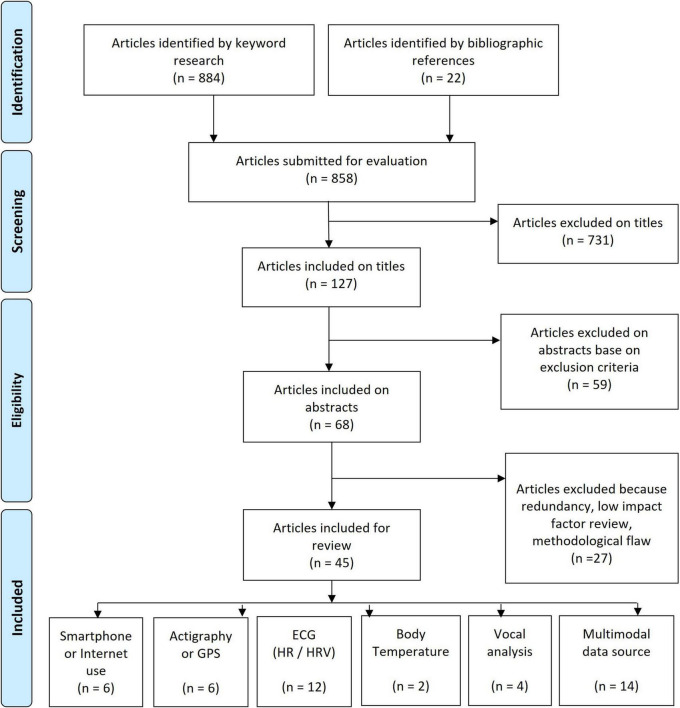
PRISMA diagram.

We conducted a review of the literature by updating a previous article (4) covering the literature from 2010 to February 2017. The present review was conducted by querying the PubMed database between February 2017 and November 2021 for titles with the terms [computer] OR [computerized] OR [mobile] OR [automatic] OR [automated] OR [machine learning] OR [sensor] OR [heart rate variability] OR [HRV] OR [actigraphy] OR [actimetry] OR [digital] OR [motion] OR [temperature] with each of the terms AND each of the following: [mood], [bipolar], [depression], [depressive], [manic], [mania]. For studies published before 2017 see Bourla et al. (4). Article selection can be seen in the PRISMA diagram (Figure 2).

Exclusion criteria were:

• Reviews and meta-analyses
• The use of digital phenotype for evaluating treatment response
• The use of digital phenotype for evaluating interventions
• The use of medical equipment for HRV/HR assessment (only smartphone sensors)
• Therapeutic interventions
• Postpartum depression or pregnancy studies
• Child or adolescent studies
• Age above 65 years
• Comorbidities (e.g., post-stroke depression, chronic obstructive pulmonary disease, Human Immunodeficiency Virus, cardiosurgical patients)
• Multimodal evaluation with blood or imagery biomarkers
• Study protocols
• Case reports
• Ecological Momentary Assessment (EMA) or self-rating questionnaires or self-reports not associated with digital phenotype.

## Results

Some studies use multiple sensors at the same time (multimodal data source), while other studies focus specifically on one type of sensor (unimodal data source). Forty-five articles were included and classified by data source: Smartphone and Internet use, actigraphy and GPS, electrocardiogram (ECG), voice analysis, body temperature and multimodal data source.

### Unimodal data source

Results using a unimodal data source are summarized in Table 1.

**TABLE 1:** Unimodal data source.

	Study (year)	Disorder	*n*	Questionnaire	Features	Accuracy	Sensitivity	Specificity
Smartphone	Safa et al. (5)	MDD	1023	LIWC	Twitter posts and bio-text	83–91%	n.c.	n.c.
	Yue et al. (7)	MDD	79	PHQ9	Metadata of internet traffic	80%	n.c.	n.c.
	Gillett et al. (9)	BD/BPD/HC	55	QIDS	Phone calls and SMS messaging	n.c.	n.c.	n.c.
	Razavi et al. (10)	BD	412	BDI – II	Phone use (calls and text messages)	76–81%	n.c.	n.c.
	Islam et al. (6)	MDD	7145 uc	LIWC	Comments on Facebook	n.c.	n.c.	n.c.
	Zulueta et al. (11)	BD	16	HDRS/YMRS	Metadata of keystroke entry with accelerometer	n.c.	n.c.	n.c.
Actigraphy	Difrancesco et al. (14)	MDD/GAD	359	Deutsch 30-IDS	Sleep parameters	n.c.	n.c.	n.c.
	Minaeva et al. (16)	MDD	179	IDSR/CIDI/BDI-II	Global activity	n.c.	n.c.	n.c.
	Jakobsen et al. (17)	BD	55	MADRS	Global activity	n.c.	82%	84%
	Kaufmann et al. (20)	BD	131	YMRS	Sleep parameters	n.c.	n.c.	n.c.
	Merikangas et al. (13)	BDI/BDII MDD	242	PHQ9	Global activity	n.c.	n.c.	n.c.
	Tonon et al. (15)	BD	15	YMRS	Global activity	n.c.	71%	100%
	Zhang et al. (12)	MDD	308	PHQ8	Bluetooth features	n.c.	n.c.	n.c.
HR or HRV	Gregório et al. (33)	HP/BD	36	MINI/BrMaS	Heart parameters	n.c.	n.c.	n.c.
	Ortiz et al. (30)	BDI/BDII	53	IBI/SADSL/MADRS/ YMRS	Heart parameters	n.c.	n.c.	n.c.
	Brugnera et al. (28)	HP	65	BDI II	Heart parameters during stress protocol	n.c.	n.c.	n.c.
	Byun et al. (25)	HP/MDD	78	HAMD	Heart parameters during stress protocol	74%	73%	75.6%
	Byun et al. (26)	MDD	66	HAMD	Heart parameters during stress protocol	70%	64%	76%
	Hartmann et al. (24)	HP/MDD	127	HDRS 17	Heart parameters	n.c.	n.c.	n.c.
	Lesnewich et al. (23)	HP	152	BDI II	Heart parameters	n.c.	n.c.	n.c.
	Faurholt-Jepsen et al. (17)	BD	16	HDRS17/YMRS	Heart parameters	n.c.	n.c.	n.c.
	Wazen et al. (31)	BD1	19	MINI/BRMS	Heart parameters during hospitalization	n.c.	n.c.	n.c.
	Carnevali et al. (21)	HP	42	RRS	Heart parameters	n.c.	n.c.	n.c.
	Chen et al. (17)	HP/MDD	80	No	Heart parameters during stress protocol	n.c.	n.c.	n.c.
	Kuang et al. (27)	MDD	76	PID	Heart parameters during stress protocol	86.4%	89.5%	84.2%
Temperature	Ma et al. (35)	MDD/SR	62	HAMD17/PHQ9/HAMA	Temperature during treatment	n.c.	n.c.	n.c.
	Kim et al. (36)	MDD	67	HAMD	Electrodermal activity during stress protocol	74%	74%	71%
Voice	Shin et al. (37)	MDD	93	MINI/BAI/HDRS/ BIS/PHQ9	Voice characteristics	n.c.	65.6%	66.2%
	Weiner et al. (39)	BP	56	YMRS/QIDSC16	Voice characteristics	83–86%	n.c.	n.c.
	Weintraub et al. (40)	BP	123	LIWC	Emotional expression	75.2–81.8%	70%	80%
	Zhang et al. (38)	MDD	n.c.	PHQ9	Voice characteristics in audio files	n.c.	n.c.	n.c.

BAI, Beck Anxiety Inventory; BDI II, Beck Depression Inventory; BP, Bipolar Disorder; BPI, Bipolar Disorder Type I; BPII, Bipolar Disorder Type II; BrMaS, Bech-Rafaelsen Mania Scale; GAD, Generalized Anxiety Disorder; HAMD17 or HDRS, Hamilton Depression Rating Scale; HAMA, Hamilton for Anxiety; HP, Healthy Patient; IDSR, Inventory of Depressive Symptomatology (self-report); LIWC, Linguistic Inquiry and Word Count; MADRS, Montgomery Asberg Depression Rating Scale; MDD, Major Depressive Disorder; MINI, Mini-International Neuropsychiatric Interview; PHQ9, Patient Health Questionnaire 9; QIDS, Quick Inventory Depression Scale; uc, user comments; n.c., not communicated; SR, Suicidal Risk; SA, History of Suicidal Attempt; w, week; YMRS, Young Mania Rating Scale.

#### Internet use

Safa et al. (5) and Islam et al. (6) explored the potential of linguistic approaches on Twitter and Facebook. They focus on tweets or comments, and use n-gram language model, or Linguistic Inquiry and Word Count (LIWC), based on the relationships between patterns of language, first person pronouns, anger words, various negative emotions and mental disorders. They show 91 and 83% accuracy in predicting depressive symptoms, respectively. Linguistic data from Facebook comments provides the highest accuracy. Yue et al. (7) explore metadata of internet traffic on smartphones for depression screening. They develop techniques to identify internet usage sessions and create their own algorithm to predict depression correlating with the Patient Health Questionnaire 9 (PHQ9). The internet traffic data was divided into application categories (e.g., mail, social, video, audio, game, shopping, study) with a focus on usage (total duration, number of sessions, duration during morning afternoon, and night, and screen on-off events) and they achieve a specificity up to 77% for depression prediction.

#### Smartphone use

##### Depressive disorders

Opoku Asare et al. (8) studied 629 individuals assessing multiple features: battery consumption, time zone, time stamped data, foreground app usage, internet connectivity, screen lock and unlock logs with demographic information and self-reports (PHQ8). They find a positive correlation between screen status-normalized entropy (defined as the degree of variability, complexity and randomness in behavior states, e.g., disconnection and connection states, frequency of use, etc.) and depression. But they find no exploitable association between other screen, app, and internet connectivity features. Using their best supervised machine learning, they achieved accuracy of up to 92.51%.

##### Bipolar disorder

Gillett et al. (9) showed a significant interaction between a bipolar disorder population and smartphone use. A negative correlation is highlighted between total outgoing call frequency, total duration, total outgoing SMS messaging frequency and bipolar depressive episodes. Razavi et al. (10) confirmed that call durations and number of text messages had a negative correlation with depressive symptoms. Furthermore, using machine learning (random forest classifier), they found an accuracy of up to 81.1% for diagnosing bipolar depression. Zulueta et al. (11) demonstrate a significant positive correlation between accelerometer displacement, average interKEY delay, session count and autocorrect rate and depressive symptomatology using the Hamilton Depression Rating Scale (HDRS).

#### Actigraphy or GPS use

##### Depressive disorders

Zhang Y. et al. (12) extracted 49 Bluetooth features including periodicity and regularity of individuals’ life rhythms using Nearby Bluetooth Device Count (NBDC; detected by Bluetooth sensors in mobile phones). During a two-year follow up study with 308 patients, they tried to correlate these features with a bi-weekly PHQ8 questionnaire assessing for depression. They show that before a depressive episode, several changes were found in the preceding 2 weeks of Bluetooth data (the amount, the variance and the periodicity decreased and NBDC sequence became more irregular). Merikangas et al. (13) highlight a direct and positive correlation of sleep and energy (assessed by minute-to-minute activity counts from an actigraphy device worn on the non-dominant wrist for 2 weeks) with mood in a large nested-case control study. They found a unidirectional association between motor activity and subjective mood level and a bidirectional association between motor activity and subjective energy level and sleep duration. Difrancesco et al. (14) obtained similar results, with a negative correlation of gross motor activity (GMA) and sleep parameters with depressive symptoms. Tonon et al. (15) demonstrate the reliability of using an actigraphic strategy for evaluating the intensity of depression. They show a sensitivity up to 71% and a specificity up to 100% for evaluating melancholia in depressed patients, since nocturnal activity was significantly higher in non-melancholic patients. Minaeva et al. (16) explore a predictive model based on actigraphy and Experience Sampling Methodology (ESM). The actigraphy model was provided by the GMA and time and maximal activity level across the 24-h period. The ESM model has a fixed design, with questionnaires including items on current mood states, social interactions, daily experiences and behaviors. They found reasonable discriminative ability for the actigraphy model alone and excellent discriminative ability for both the ESM and the combined-domains model (actigraphy + ESM). These results were based on a strong correlation between depression and lower levels of physical activity.

##### Bipolar disorder

Jakobsen et al. (17) used several machine-learning techniques using actigraphy and observed an 84% accuracy, sensitivity and specificity when differentiating between depressed bipolar patients and healthy individuals. Jacobson et al. (18) re-analyzed Jakobsen’s data using novel methods and their machine-learning algorithm correctly predicted the diagnostic status 89% of the time. These results remain inconsistent, as observed by Freyberg et al. (19) since they found no difference between healthy controls and younger bipolar patients in activity energy expenditure, suggesting that these outcomes could progress along with disease duration. Kaufman et al. (20) explored several sleep features using night actigraphy: total sleep time, waking after sleep onset, percent of sleep and number of awakenings. Using a ML algorithm (LASSO regression) they found that none of those features differ between bipolar patients and healthy controls. However, there is considerable variability among those with bipolar disorder. Zulueta et al. (11) demonstrate a significant correlation between accelerometer displacement and HDRS or YMRS during depressive or manic episodes.

#### Heart rate/heart rate variability

##### Depressive disorders

Carnevali et al. (21) have published a long-term follow-up study of 3 years, assessing HRV at several points (T0, 13th and 34th month). They find that resting HRV is negatively correlated with both rumination and depressive symptoms. They suggest a link between HRV at T0 and the evolution from rumination and depressive symptoms at month 13. They also conclude that a low vagal tone is a characteristic of depressive symptoms, which is consistent with Chen et al. (22), who have suggested the implication of an over-activated parasympathetic nervous system under long-term depression. Thus, a negative correlation is usually found between HRV and diagnosis of depression. But use of current antidepressants can turn this correlation into a positive one as Lesnewich et al. demonstrated in (23). Hartmann et al. (24) demonstrate that HRV could be used as a unique biomarker which could vary with the depressive symptomatology and be correlated with symptom severity. Byun et al. (25) used machine learning on 20 HRV indices and achieved an accuracy of 74.4%, a sensitivity of 73%, and a specificity of 75.6% for depression detection. In another study (26), they found a similar result, proving that entropy features of HR are lower in depressive patients in stress conditions. All those results are in line with Kuang et al. (27) who obtained an accuracy of 86.4%, sensitivity of 89.5%, and specificity of 84.2% for depression. Brugnera et al. (28) showed a significant and positive relationship with a higher resting-state HRV, but confirmed a blunted reactivity to the stress protocol. They suggest that healthy individuals with higher depressive symptoms have atypical cardiovascular responses to stressful events. Despite these results, Sarlon et al. (29) have not found any interaction between HRV and depression severity.

##### Bipolar disorder

Ortiz et al. (30) showed a positive correlation between reduction of HRV and illness duration, number of depressive episodes, duration of the most severe manic/hypomanic episodes, co-morbid anxiety disorder and family history of suicide. Wazen et al. (31) observed an increase in HR and decrease in HRV in mania relative to euthymia. Faurholt-Jepsen et al. (32) found an increase of 18% of HRV in mania state compared with a depressed state, but no significant HRV difference has been found between euthymia and the depressed stage in bipolar disorder. Gregorio et al. (33) showed non-linear HRV dynamics consistent with high sympathetic heat modulation with less vagal modulation compared to healthy controls. They show a possible reduction of sympathetic modulation after treatment, with an increased vagal or parasympathetic modulation.

#### Body temperature

Lorenz et al. (34) first showed no significant temperature difference between mood disorders and healthy control groups, but after excluding antidepressant-medicated participants, they found that the depression group has lower skin temperature amplitude and a less stable skin temperature rhythm. Ma et al. (35) find a peripheral body temperature rhythm higher in major depressive disorder patients than in healthy controls. They highlighted the existence of phase delay of temperature that was greater in mood disorder patients with suicidal risk than those without suicidal risk and healthy controls. The same results can be found with patients before treatment introduction. They suggest that the body temperature anomalies can diminish with the improvement of depressive symptoms after the treatment with antidepressants. Kim et al. (36) used a machine learning approach to analyze an electrodermal activity data set and predict MD diagnoses during a stress protocol. They observed 74% accuracy and sensitivity and 71% specificity for depression.

#### Vocal recording

##### Depressive disorders

Shin et al. (37) used machine learning on data extracted from semistructured interview recordings. Previous research showed that depressed patients had simpler, lifeless voices with lower volume. Their movements of the vocal tract were slow and they spoke in low voices. Thus, the features extracted are from four aspects: namely glottal, tempo-spectral, formant, and other physical features. Glottal features included information about sound articulation with the vocal cords, obtained by parameterizing each numeric after drawing a waveform. They used it for calculating three parameters: the opening phase, closing phase, and closed phase. They extracted tempo-spectral features with the help of “librosa,” an audio processing toolkit. Temporal features refer to the time or length of the interval when participants continue an utterance. For spectral features, they used averaged spectral centroid, spectral bandwidth, roll-off frequency, and root mean square energy. Formant features are phonetic information obtained through linear prediction. The formant was considered as the resonance of the vocal tract and as the local maximum of the spectrum. The first to third formants were exploited with their bandwidths. Other physical attributes were obtained as the mean and variance of pitch and magnitude, zero crossing rate (which indicated how intensely the voice was uttered), and the voice portions (which indicated how frequently they appeared). Silence was represented by frames with zero crossing rates below the average. This study was conducted on 93 patients in three different groups: non-depressed, major depression, and minor depression. The minor depression group had the lowest voices and more pitch changes during speech. The major depression group was between the non-depressed and the minor depression group. A multilayer processing method achieved 65.9% accuracy, 65.6% sensitivity, and 66.2% specificity for distinguishing depressed people from healthy controls. Zhang L. et al. (38) were able to predict the PHQ9 score with an AUC of 0.825 with features extracted from recorded audio of depressed subjects.

##### Bipolar disorder

Weiner et al. (39) applied machine learning on verbal fluency tasks (letter, semantic, free word generation, and associational fluency data) to classify two distinct acute episodes in each of 56 patients as manic, mixed manic, depressive, or mixed depressive. They used a two-step procedure with a first one beginning by selection of single words using a voice activity detection algorithm. Then, speech features were calculated for each word. The word detection algorithm used the energy of the audio signal to analyze the temporal and spectral features. Then, they used the Camacho SWIPE algorithm according to a spectral matching procedure, which analyses signal intensity, zero crossing rate, spectral strength, and finally permitted detection of single words. Speech feature estimation was based on the estimation of specific features related to prosody and voice quality. Pauses calculated between words, word length and estimating F0 (pitch) dynamics (temporal windows of 10 milliseconds) were obtained with the SWIPE algorithm and supplied the prosodic features. For each word, they supplied estimates of F0, median, median absolute deviation. Then they extended the use of these features to all the voiced segments within each word and finally they reported the resulting features as amplitude, duration and tilt (mean of amplitude and duration). Voice quality was obtained by estimating the long-term muscular setting of the larynx and vocal tract that deviated from the neutral point which was calculated with the help of a DYPSA algorithm. They highlighted that in the mixed manic and manic groups, voice quality patterns were elevated in subjects with a high score in the Quick Inventory of Depressive Symptomatology (QIDS-c16) questionnaire. Higher score on the Young Mania Rating Scale (YMRS) questionnaire was correlated with higher median pitch, with a higher variability as expressed by the dispersion of voiced sound fundamental frequency, and with higher tilt. Given these results, they selected features for a mixed versus non-mixed and depression algorithm detection. They achieved an accuracy of 84% for discriminating depression from mixed depression, 86% for discriminating hypomania from mixed hypomania. They suggest that vocal features *via* verbal fluency tasks could be reliable biomarkers and help to improve diagnostic accuracy. Weintraub J. et al. (40) also used machine learning to achieve an algorithm with an accuracy of at least 75.2% for detecting high or low expressed mood.

Several studies tried to demonstrate a new correlation between DP and mood disorders, while others tried to prove the reliability of predictive models using different machine learning algorithms based on the previous correlations. Only a few focused on specific symptoms of mood disorders.

### Multimodal data source

Results from multimodal data sources are summarized in (Table 2).

**TABLE 2:** Multimodal data source.

Study (year)	Disorder	*n*	Smart-phone	GPS or actigraphy	HR – HRV	Body tempe-rature	Voice	Light exposure	Scale	Accuracy
Bai et al. (42)	MDD	334	Yes	Yes	Yes	Yes	Yes	No	PHQ9	76.67%
Meyerhoff et al. (41)	MDD/GAD/SAD	282	Yes	Yes	Yes	No	No	No	PHQ8/DAS7	n.c.
Nickels et al. (44)	MDD	415	Yes	Yes	No	No	No	Yes	PHQ9	n.c.
Opoku Asare et al. (8)	MDD	629	Yes	No	No	No	No	No	PHQ8	98%
Rykov et al. (43)	MDD	267	Yes	Yes	Yes	No	No	No	PHQ9	80%
Sarlon et al. (29)	MDD	89	No	No	Yes	Yes	No	No	BDI II	No
Di Matteo et al. (48)	Gen pop	112	Yes	Yes	No	No	Yes	Yes	PHQ8	n.c.
Jacobson and Chung, (49)	MDD	31	Yes	Yes	Yes	No	No	Yes	PANAS-X	n.c.
Narziev et al. (50)	MDD	20	Yes	Yes	Yes	No	No	Yes	PHQ9	96%
Freyberg et al. (19)	BD	60	No	Yes	Yes	No	No	No	HDRS17	n.c.
Pedrelli et al. (45)	MDD	31	Yes	Yes	Yes	No	No	No	HDRS17	n.c.
Cho et al. (47)	MDD/BDI/BDII	55	Yes	Yes	Yes	No	No	Yes	Mood chart app	85/94%
Jacobson et al. (46)	BDII	15	No	Yes	No	No	No	Yes	MADRS	84%
Lorenz et al. (34)	MDD	242	No	Yes	Yes	Yes	No	No	CES D score	n.c.

BDI, Bipolar Disorder Type 1; BDII, Bipolar Disorder Type 2; BDI II, Beck Depression Inventory; CES D, Centre for Epidemiological Studies-Depression; DAS7, Dyadic Adjustment Scale; GAD, General Anxiety Disorder; Gen pop, General population; HDRS, Hamilton Depression Rating Scale; HP, Healthy People; MADRS, Montgomery Asberg Depression Rating Scale; MDD, Major Depressive Disorder; n.c., not communicated; PANAS-X, Positive and Negative Affect Schedule Expanded; PHQ9, Patient Health Questionnaire 9; SAD, Social Anxiety Disorder; w, week.

Meyerhoff et al. (41) studied a cohort of 282 healthy individuals who used wearable devices measuring steps, energy level, HR, and sleep change and trained a supervised machine learning algorithm to study the interaction between those passive data and a PHQ8 questionnaire. They observed changes in GPS features, exercise duration, and use of active apps before the rise of depressive symptoms, suggesting a directional correlation between changes in behaviors and subsequent changes in depressive symptoms. Bai R. et al. (42) recruited 334 patients with a major depressive disorder to a study using an app called “Mood Mirror” that allows active data and passive data collection (phone and wearable wristband) in order to classify patients between several mood states: steady remission, mood Swing (drastic or moderate), and steady depressed. They tested several combinations of data in order to achieve the best classification. The best features were passive data (1 feature from phone usage and 3 from the wearable) and achieved over 75% accuracy.

Rykov et al. (43) recruited 267 healthy people and used wearables to record physical activity, sleep patterns, circadian rhythms (CRs) for step usage, HR, energy expenditure and sleep data. They used supervised machine learning and found an accuracy of 80%, a sensitivity of 82%, and a specificity of 78% for detection of depression, but only in subsamples of depressed and healthy participants with no risk of depression (no or minimal depressive symptoms). Apart from this, the ability of the digital biomarkers to detect depression in the whole sample was limited. Nickels et al. (44) recruited 415 individuals (around 80% with MDD) and created a large specific operating system that recorded accelerometer, ambient audio, phone information, barometric air pressure, battery charge, Bluetooth, light level, network, gyroscope, physical activity level, phone calls, ping, proximity, screen state, step count, text messages, volume, and Wi-fi network. Using a subset of 34 DP features, they found that 11 features showed a significant correlation with PHQ9. They found that a more negative sentiment of the voice diary, obtained from a derived measure from a sentiment classification algorithm, is associated with a higher PHQ9 score. Moreover, bad self-reported sleep quality, higher ambient audio level, letting the phone ring for longer periods until the call was missed, fewer different locations visited in a given week, fewer words spoken per minute, longer duration of voice diary, lower weekly mean battery percentage, the number of emojis in outgoing text messages, receiving or making more phone calls per week, and less variability in where participants spent time were correlated with higher PHQ9 score. They achieved a logistic regression model resulting in a 10 fold cross-validated mean AUC of 0.656 (SD 0.079).

Pedrelli et al. (45) observed that it was not possible to determine if one modality (smartphone, wearable or both) could outperform the others. They highlighted that the most predictive features were related to phone engagement, activity level, skin conductance and HRV, but stated that further studies are needed to increase strategy accuracy. Jacobson et al. (46) conserved a rate of accuracy to predict depression with only a 1 week recording study with 15 participants with MDD. They combined only two assessing tools with actigraphy, which records continuous movement, to estimate global activity and with ambient light exposure to estimate social activity. A deep neural network combined with SMOTE class balancing technique achieved an accuracy of 84%, a sensitivity of 82%, and a specificity of 84%. A prospective observational cohort study was performed by Cho et al. (47) on 55 patients with MDD and bipolar disorder type 1 and type 2 during 2 years using a smartphone-based EMA and a wearable activity tracker (Fitbit). They processed the digital phenotypes into 130 features based on circadian rythms (e.g., steps before bedtime, light exposure during daytime, and HR amplitude) and performed mood classification using a random forest algorithm.

Di Matteo et al. (48) designed an Android app to collect periodic measurements including samples of ambient audio, GPS location, screen state, and light sensor data during a 2-week observational study. They found good accuracy with an AUC of 0.64.

Jacobson et al. (49) used passive sensor data, including GPS, location type based on the Google Places location type, local weather information (temperature, humidity, precipitation), light level, HR information (average HR and HRV), and outgoing phone calls, and used machine learning algorithms to correlate these data with a dynamic mood assessment using EMA. They found good accuracy, with predicted depressed mood scores that were highly correlated with the observed depressed mood scores from the models.

Narziev et al. (50) designed a Short-Term Depression Detector using EMA and various passive sensors available on a smartphone (phone calls, app usage, unlocked state, stationary state, light sensor, accelerometer, step detection) and on a smartwatch (HRM, accelerometer). They used support vector machine and random forest models to achieve group classification with an accuracy of 96.00%.

## Discussion

### Principal results

Digital phenotypes compute the clinical characteristics specific to various mental states, sometimes with better precision than a clinician can achieve, and with the possibility of doing it remotely. Table 3 summarizes the most relevant features according to this review and our previous one (4).

**TABLE 3:** Digital phenotype of features relevant to mood disorders.

	MDD or bipolar depression	Mania
Actigraphy	Decreased daytime activities Decreased walking Nearby Bluetooth Device Count: Number, variance and periodicity are decreased and NBDC sequence becomes more irregular Nocturnal activity is significantly higher in non-melancholic patients Unidirectional association between motor activity and subjective mood level and a bidirectional association between motor activity and subjective energy level or sleep duration. Negative correlation between gross motor activity and sleep parameters with depressive symptoms Lower level of activity Late start of activities Peak activity at noon Poor evening activities	Increase in activities Increase in number of locations
HR and HRV	Severity-dependent decrease in HRV Increase in low-frequency HRV and low-frequency/high-frequency ratio Decreased high-frequency HRV Higher heart rate and lower HRV in bipolar than in unipolar depression Increase in the low-frequency/high-frequency ratio and decrease in high-frequency HRV in BPII Resting HRV is negatively correlated with both rumination and depressive symptoms Reduction of HRV is correlated with illness severity	Decreased RR interval (increased HR), variance, low-frequency HRV, and high-frequency HRV; Increase in the low-frequency/high-frequency ratio Increased and decreased HRV were both found
Temperature	Decrease in temperature Night-time temperature increase Decreased amplitude Phase advance Depression group has a lower skin temperature amplitude and a less-stable skin temperature rhythm Body temperature rhythm higher in major depressive disorder patients than healthy Body temperature anomalies can diminish with the improvement of depressive symptoms	n.c.
Smartphone	Decrease in smartphone use (number of SMS messages, number of calls) Change in the duration of calls Linguistic data: patterns of language, first person pronouns, anger words, various negative emotions expressed on Twitter or Facebook Internet traffic data (mail, social, video, audio, game, shopping, study): total duration, number of sessions, duration in morning, afternoon, and midnight, and screen on-off events Smartphone use: outgoing call frequency, total duration, total outgoing SMS messaging frequency, call durations, number of text messages, average interKEY delay, session count and autocorrect rate Positive correlation between screen status-normalized entropy and depression	Number of calls increased; Increased call duration; Number of messages increased
Voice	Increased response latency; Number of breaks and length of breaks increased	Reduced number of breaks Increased verbal fluency
Multimodal	The most predictive features were related to phone engagement, activity level, skin conductance, and heart rate variability	

MDD, Major Depressive Disorder; HRV, Heart Rate Variability; HR, Heart Rate; SMS, Short Message Service; NBDC, Nearby Bluetooth Device Count.

The unimodal data source type is the first and the most common type of study. As we can observe in Table 1, we have as many studies focused on the new correlation demonstration as new predictive models with different levels of precision. It can be explained by a faster and easier protocol, with more innovative possibilities. Results in unimodal data sources have an accuracy around 75% with a sensitivity range between 64 and 91%, and a specificity between 66.2 and 100%. Studies focusing on smartphone use are the most dynamic unimodal approach, providing disruptive ways to a better understanding of mood disorders. The tools which are used for that purpose are multiple: call frequency, SMS message frequency, SMS message length, keystroke entry date, accelerometer displacement, activity tracker *via* Bluetooth, usage sessions from internet traffic, and linguistic analyses on Twitter and Facebook posts with the help of EMA provided by smartphone apps. In most cases, applications are developed specially for each study. ECG recording is the most common type of wearable found in this review. Features were Resting HR, HRV, Respiration Rate, high-frequency HRV, low-frequency HRV, and root mean square of the successive differences, with experimental exposition using a protocol for assessing the autonomic responses to stress and recovery. Body temperature recording is also a promising source. This extracts global body temperatures using a Holter monitor that detects 24-h peripheral body temperature. Several studies combined temperature with actigraphy, making it possible to categorize body temperature throughout the day’s phases, and adjust the amplitude with activity. While promising, voice analysis still remains one of the poorest study domains. The data provided come from speech text provided by smartphone and use of acoustic data automatically recorded. Other metrics can also be calculated: total speech activity, the proportion of speech during a day, analysis of voice and linguistic patterns, GPS location provided by environmental sounds, and verbal fluency tasks (letter, semantic, free word generation, and associational fluency). Activity of humans in daily life is often represented through GPS location or actigraphy on wearables that can be used to study physical activity, recording GMA, and sleep quality with CR. This technique helps us to understand the link between state of health, activity and sleep conditions. They try to implement reliable values such as total sleep time, wake after sleep onset, percent sleep and number of awakenings, sleep latency, sleep efficiency, and relative amplitude between daytime and night-time activity.

The multimodal data source approach uses combinations of the research presented from the unimodal data. We can observe that in most cases, it is focused on the development of new predictive models for diagnosing mood disorders. Therefore, in most cases it uses machine learning for training new helpful algorithms for increased diagnostic accuracy. Different models are proposed to achieve this goal. However, multimodal data sources, despite the promising possibilities of this technique, have hardly succeeded in demonstrating new disruptive comprehension of the DP of mood disorders.

Beyond the aspects mentioned in this article, the digital phenotype offers interesting perspectives for treatment and clinical research, in particular for the dimensional approach to mental disorders. Because mood disorders manifest themselves in a heterogeneous way on the different components of the mind, psychiatric classifications have often been criticized, particularly for their validity, and more contemporary approaches attempt to increase their reliability by using more integrative approaches, the most successful of which is the RDoC matrix (51). This model proposed by the NIMH offers a relevant framework to better exploit the links existing between clinical and biological markers from a dimensional perspective. The numerous passive and active markers of DP make it possible to collect information specifically related to each clinical dimension (emotional and affective, cognitive, conative, and physical) and therefore to propose a more detailed evaluation of semiology. In this perspective, Torous et al. (3) state that it will thus be possible to collect preclinical information at the community level, thus establishing a reference level of the psychological dimensions of the general population and to compare them with the pathological variations observed in clinical populations. We suppose that it will be possible to determine predictive patterns for the occurrence of a clinical episode; in short, to make predictions.

From a therapeutic point of view, use by clinical dimension therefore makes it possible to offer, in real time, more specific and therefore personalized interventions. For example, in a recent article we have listed the interventions more specific to the conative dimension (which refers to goal-directed behavior intentionally based on motivational factors), which can be provided in support of active and passive DP data (52).

Finally, as mentioned above, DP can be an effective tool for bias mitigation. Indeed, the processes related to decision-making can benefit at several levels from the data collected, both active and passive. For illustration, the passive collection of data, less subject to the subjectivity of the patient and the clinician, makes it possible to have a more efficient control of certain cognitive human constraints (recall bias for example).

### Limitations

Some limitations have been found in the review. The majority of apps are available on Android, but only a few on iOS. That means that data collected could introduce a new type of bias based on social grounds. A significant number of studies show an over-representation of the female population. Large cohort studies remain the exception to the rule, and the ability of digital phenotype to be widely and easily deployed has remained untapped. That could be explained by the legal procedures for protection of privacy, which differ significantly between countries. Therefore, there is an overrepresentation of the American or Asian population in digital phenotype studies.

The majority of the recent studies focused on smartphone use, HRV, GPS and multiple wearable strategies. Multimodal strategies can be observed to present better reliability than the unimodal approach. Questionnaires used by studies are different, making comparisons more difficult, although use of the PHQ9 (or PHQ8) questionnaire is common. Further exploration could be achieved through other technologies, bringing more accuracy with less recording, which is logically more efficient.

### Comparison with prior work

Most authors recommend the use of passive data preferentially to active data in the context of bipolar disorder because this type of automatically generated data makes it possible to limit bias and limit the feeling of intrusion that self-questionnaires can cause (especially if they must be filled in regularly or if they appear in a “pop-up”). Some authors emphasize the limitations of the actual DSM-5 approach, which is based on clinical statistical observation. As reported above, the digital phenotype provides new insight into the classification of disorders from a behavioral perspective.

The concept of a digital phenotype, which is materially supported by a technological tool that is theoretically accessible at any time, thus makes it possible to track an individual’s behavioral change processes over a more sustained period of time compared to periodic visits to the practitioner (53). However, it is not recommended for all clinical approaches and as reported by Patoz et al. (54), many studies suggest that these applications would be more appropriate for mild and moderate stages of depression. Indeed, their use in severe mood disorders is potentially limited.

We have also emphasized the appeal of passive data, which encourages the involvement and commitment of subjects more easily; for example, it requires little effort to make an inventory of one’s symptoms. O’Brien and Toms (55) point out that engagement is not a static process, but a multi-stage process: one starts at the point of engagement, then comes a period of engagement, and finally it is possible to encounter a point of disengagement and a period of re-engagement. Finally, in the absence of symptoms, the feeling of need for care may diminish and thus cause the subject to disengage. These tools allow for a more detailed and personalized behavioral follow-up, and therefore to propose effective corrective actions, such as feedback screen for promoting the reward dimension (56).

## Conclusion

Ultimately, it appears that the digital phenotype of a pathology is the computer translation of objectifiable signs of mental illness, and it should therefore be understood as a means of strengthening the observation capacities of psychiatrists. Regarding depressive disorders, the main elements are the decrease in functional and biological parameters (decrease in activity and walking, decrease in the number of calls and SMS messages, decrease in temperature and HRV) while the manic phase results in the reverse phenomenon (increase in activity, number of calls, and HRV) as one would expect. As of now, most of the studies have focused on one tool, with significant accuracy. But there still remains lack of evidence of the usability of these technologies for long-term follow-up.

## Data availability statement

The original contributions presented in the study are included in the article/supplementary material, further inquiries can be directed to the corresponding author.

## Author contributions

AB and SM: conceptualization. AB and RM: methodology. FF and BM: validation. AO, AB, and RM: writing – original draft preparation. AB, AO, and VA: writing – review and editing. BS and OB: visualization. SM and AB: supervision. FF, OB, and BM: project administration. All authors have read and agreed to the published version of the manuscript.

## Conflict of interest

The authors declare that the research was conducted in the absence of any commercial or financial relationships that could be construed as a potential conflict of interest.

## Publisher’s note

All claims expressed in this article are solely those of the authors and do not necessarily represent those of their affiliated organizations, or those of the publisher, the editors and the reviewers. Any product that may be evaluated in this article, or claim that may be made by its manufacturer, is not guaranteed or endorsed by the publisher.
